# Treating hepatocellular carcinoma with ^90^Y-bearing microspheres: a review

**DOI:** 10.7603/s40681-016-0019-z

**Published:** 2016-12-06

**Authors:** Te-Chun Hsieh, Yu-Chin Wu, Shung-Shung Sun, Kuo-Yang Yen, Chia-Hung Kao

**Affiliations:** 1 Department of Nuclear Medicine and PET Center, China Medical University Hospital, No. 2, Yuh-Der Rd., North Dist., Taichung, 404 Taiwan; 2 Department of Biomedical Imaging and Radiological Science, China Medical University, 404 Taichung, Taiwan; 3 Department of Nuclear Medicine, National Taiwan University Hospital Hsin-Chu Branch, Hsin-Chu Branch, No. 25, Ln. 442. Sec. 1, Jingguo Rd., East Dist.,, Hsinchu City, 300 Taiwan; 4 School of Medicine, China Medical University, 404 Taichung, Taiwan

**Keywords:** Hepatocellular carcinoma, ^90^Y microspheres, Selective internal radiation therapy, ^99m^Tc macroaggregated albumin, Transarterial chemoembolization, External beam radiation therapy, Radiation lobectomy

## Abstract

Hepatocellular carcinoma (HCC) is a disease usually diagnosed in its advanced-stage, and is frequently not amenable to curative surgical treatment. Also, HCC is resistant to chemotherapy and less vulnerable to radiation therapy compared to normal hepatic parenchyma. Both of these facts render the efficacy of adjuvant and palliative treatments problematic. Selective internal radiation therapy (SIRT) with ^90^Y-bearing microspheres is characterized by preferentially delivering substantially high doses of radiation to a liver tumor dose simultaneously limiting the damage to its non-tumorous cells, providing an opportunity for effective local tumor control and even tumor regression therapy. The current article reviews the specific characters, dosimetry, possible applications, and special considerations toward the pre-existing radiation therapy of ^90^Y microsphere SIRT in treating HCC.

## 1. Introduction

Hepatocellular carcinoma (HCC) is the most common primary hepatic malignancy and the third leading cause of cancer-related deaths [1]. The annual incidence of HCC is more than 1 million worldwide [2]. Approximately 80% of reported HCC cases are from East Asia and sub-Sahara Africa, which are areas greatly influenced by the prevalence of hepatitis B virus (HBV) infections [3]. Resection of the tumors, including partial hepatectomy and liver transplantation, are the only curative therapies currently available to patients suffering from HCC. However, only about 10% of patients are eligible for surgery [4]. Until now, patients with HCC have been frequently given poor prognoses because of the diagnoses of most cases is at the advanced stage [5]. In addition, HCC is resistant to chemotherapy [6] and is less vulnerable to radiation therapy compared to normal hepatic parenchyma [7]. Moreover, patients with HCC usually have liver cirrhosis or portal vein thrombosis, which both contribute to poor hepatic reserve and prevent aggressive treatments of the HCC. Hence, the estimated median survival of HCC is only 8 months [8].

Approximately 80% of the blood supply to a hepatocellular carcinoma is *via* the hepatic artery. On the other hand, 75% of the blood supply to a normal hepatic parenchyma comes from the portal vein [9-11]. Therefore, an anticancer regimen preferentially delivered *via* the hepatic artery may be advantageous in treating HCC by increasing the toxicity of the tumor and decreasing the damage done to normal hepatic tissue. As a result, transarterial embolic therapy, either with bland embolization only or combined infusion with a chemotherapeutic agent, has been widely applied as a palliative, alternative, or interim treatment for patients not eligible for curative surgery.

For patients with HCC, it is estimated that there is either a 5% or a 50% risk of radiation-induced liver disease when the whole liver is irradiated by external beam radiation with mean doses of 32 and 40 Gy, respectively [7]. Compared to external beam radiation therapy (EBRT), selective internal radiation therapy (SIRT) by transarterial infusion with ^90^Y-bearing microspheres—the high energy beta particle (maximum energy: 2.27 MeV; mean energy: 0.94 MeV) emitted by the decay of ^90^Y to ^90^Zr and minimal penetration range (average: 2.5 mm; maximum: 11 mm) in tissue—is potentially capable of delivering much higher radiation doses to tumors while the radiation exposure to the normal hepatic parenchyma remains within tolerable limits [12].

**Table 1:** 

	TheraSphere^®^	SIR-Spheres^®^
Material	Glass-based	Resin-based
Diameter	20-30 μm	20-60 μm
Activity per sphere	2500 Bq	50 Bq
Specific gravity	3.2 g/ml	1.6 g/ml
Relative embolic potential	Low	High
Relative pressure for infusion	High	Low

## 2. Comparison of microspheres

^90^Y, with a physical half-life of 64.2 hours, is produced by neutron bombardment of ^89^Y in a nuclear reactor. It decays to stable ^90^Zr with emitting high-energy beta particles. One GBq of ^90^Y delivers an absorbed dose of dose 50 Gy per kilogram of tissue. Currently there are two types of commercially available ^90^Y-bearing microspheres: glass-based (TheraSphere^®^, MDS Nordion, Ottawa, Ontario, Canada) and resin-based (SIR-Spheres^®^, Sirtex Medical Limited, Sydney, Australia) microspheres [13]. The characteristics of these microspheres are listed in Table 1.

## 3. Procedure and dosimetry

**Figure 1:**
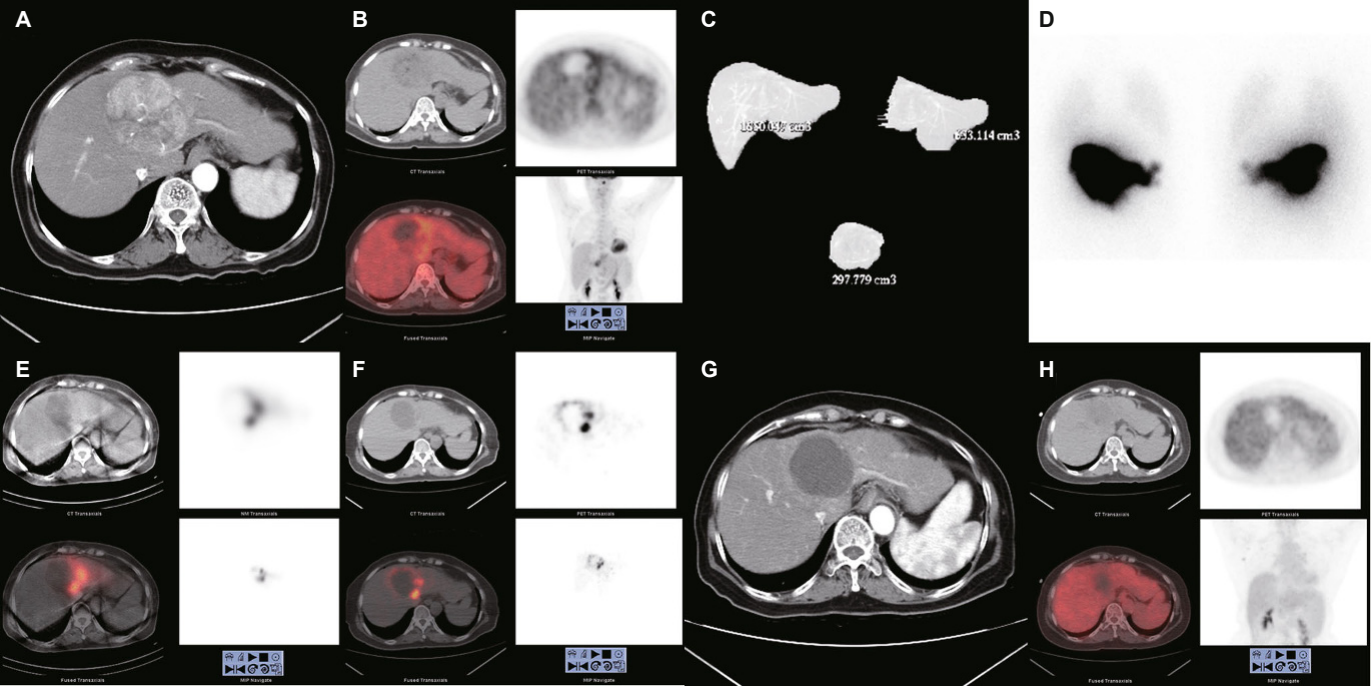

The therapy consists of two angiographic procedures. The first procedure delivers ^99m^Tc macroaggregated albumin (MAA) to the liver in order to simulate the deposition of the therapeutic microspheres that will follow. Planar and single photon emission computed tomography (SPECT) images are obtained to detect any uptake of ^99m^Tc MAA outside of the liver, particularly in the gastrointestinal tract and lungs. Gastrointestinal uptake usually can be avoided by coiling the involved artery. Hepatopulmonary shunting, if resulting in more than 30 Gy going to the lungs, is a contraindication for ^90^Y SIRT and should be carefully evaluated. The second procedure delivers ^90^Y-bearing microspheres, with the calculated radioactivity based on the prior ^99m^Tc MAA scan and other patient-specific factors, to the same liver about 1 to 2 weeks later. Images of bremsstrahlung SPECT/CT or internal pair production positron emission tomography/computed tomography (PET/CT) of ^90^Y may be acquired to confirm the distribution of the microspheres [14] (Figure 1).

The formation of arteriovenous anastomoses or shunts may occur as part of neoplastic vasculature within tumors. These shunts may allow microspheres to bypass the terminal arterioles/ capillaries of the hepatic tumor and enter the lungs, leading to radiation pneumonitis [15]. Estimating lung damage due to liver-tolung shunting can be accomplished by calculating the percentage of lung shunting based on the planar scan of chest/abdomen with 148-185 MBq of ^99m^Tc MAA injected to the hepatic arteries [16]. Previous studies have demonstrated that the lungs may tolerate up to 30 Gy with a single injection of ^90^Y microspheres and up to 50 Gy for multiple injections [15]. Hence, potential lung shunting demonstrated on ^99m^Tc MAA scan resulting in doses greater than 30 Gy should have their treatment discontinued.

As mentioned earlier, 40-Gy whole-liver external beam radiation has been found to demonstrate a 50% incidence of radiationinduced liver disease, and the incidence steeply rises with doses above 30 Gy. However, liver doses with SIRT often exceed 50 Gy and, as found in the literature, may even exceed 100 Gy in a single infusion [17, 18]. Yet extrapolating the estimates of complication probability derived from the data of external beam radiation seems unsuitable for SIRT since the occurrence of radiation- induced liver disease is rare in the latter. The non-uniform distribution of microspheres within the liver, which leads to a very high tumor-cell-to-normal-liver-cell ratio of absorbed dose, has been proposed to explain the different effects between EBRT and SIRT [19, 20]. Currently, little is known about the maximum tolerable dose of non-tumor liver parenchyma in SIRT. Although there have been recommendations to set non-tumor dose limits as low as 70 Gy in non-cirrhotic livers and 50 Gy in cirrhotic livers [21], these limits are still arbitrarily defined and need to be confirmed in prospective studies [22].

Based on the partition model, the survival of HCC patient is better when the absorbed dose of SIRT in the tumor is > 120 Gy according to previous studies [23, 24].

Several methods have been used for determining HCC treatment. For resin-based microspheres, three different methods can be applied, including empirical, body surface area (BSA), and partition methods [25]. The simplest way is the first, the empirical method (Table 2), which needs only two to three parameters: the fraction of liver that is tumorous, the lung shunting fraction, and the target liver range. However, this method greatly depends on tumor load and does not consider other patient-specific factors, such as liver function reserve. It has been found to relate to an unacceptable clinical and laboratory toxicity profile and thus has been not recommended for routine use [26, 27].

The second method is based on body-surface area, or BSA. It is a semi-empirical method and has been used safely in many clinical trials. The activity is determined by the following equation:

**Activity** (GBq) = [*BSA* (m^2^) – 0.2] + (*tumor volume/total liver volume*)

The BSA method is a relatively conservative estimation of activity compared to the empirical method. The main limitation of this method is the absence of target volume in the calculation, resulting in possible under-treatment (for example, a small patient with a large liver) or over-treatment (a large patient with a small liver) [26, 28]. In addition, the differences of individual intrahepatic distribution of radioactivity in tumor and non-tumor livers are not considered, which is also disadvantage for patients with hyper- or hypo-vascular tumors [22].

The third method is the so-called partition method. This assessment partitions the radioactivities with their individual masses in the tumor and normal liver according to the ^99m^Tc-MAA SPECT and contrast-enhanced CT. Although this method is very intuitive and based on the aforementioned principle of calculating and balancing the need for minimum amount for the maximum tumoricidal effect with maximum tolerable normal liver doses, the actual execution is usually difficult and time-consuming, particularly when there are multiple small tumors throughout a liver. The dosimetry uses the medical internal radiation dose (MIRD) model. In short, the partition method provides an average of the activity per unit of tumor mass and per unit of normal liver mass respectively, which can be used for estimating average doses to deliver to the tumor(s) and to the normal liver. The calculation uses following equations:

**Table 2:** 

**Extent of disease**	
**Fraction of liver involvement**	**Base activity (GBq)**
> 50%	3
25-50%	2.5
< 25%	2
**Lung shunting**	
**Fraction of counts in the lung**	**Dose modifier**
< 10%	1.0
10-15%	10-15%
15-20%	0.6
> 20%	Do not proceed.
**Target**	
**Part of liver**	**Dose modifier**
Whole liver	1.0
Right lobe only	0.7
Left lobe only	0.3

Prescribed activity = base activity × lung shunting modifier × target liver fraction modifier.

Equation 1:

**Lung shunting fraction** = *Radioactivity counts of lung / (Radioactivity counts of lung + Radioactivity counts of liver)*

Equation 2:

***T:N*** (tumor to normal liver ratio) = (*Radioactivity counts of tumor / Mass of tumor*) / (*Radioactivity counts of normal liver / Mass of normal liver*)

Equation 3:

***Activity*** (GBq) = {*Dose to normal liver* (Gy) × [*T:N × Mass of tumor* (kg) + *Mass of normal liver* (kg)] } / [50 (Gy ∙ kg ∙ GBq^-1^) × (1 - *lung shunting fraction*)]

Equation 4:

**Dose to tumor** (Gy) = *Dose to normal liver* (Gy) × *T:N*

Equation 5:

**Dose to lungs** (Gy) = 50 (Gy ∙ kg ∙ GBq^-1^) × Activity (GBq) × *Lung shunting fraction / mass of lungs* (kg)

For glass-based microspheres, the recommended dose to the liver is between 80 to 150 Gy [29]. The calculation of activity is also based on the MIRD model. However, unlike the partition method used in resin-based microspheres, this only considers average dose to the entire affected liver—*i.e.*, both the tumorous and normal liver parts.

***Activity*** (GBq) = [*Dose* (Gy) × *Mass of affected liver* (kg)] / [50 (Gy ∙ kg ∙ GBq^-1^) × (1 – *lung shunting fraction*)]

## 4. Indication and contraindication

The use of resin-based microspheres (SIR-Spheres^®^) is approved for treatment of advanced non-operable liver cancer in the European Union (EU) as well as unresectable metastatic liver tumors from primary colorectal cancer with adjuvant intra-hepatic artery chemotherapy of Floxuridine by the Food and Drug Administration in the United States (U.S. FDA). Glass-based microspheres (TheraSphere^®^) have a Humanitarian Device Exception for the treatment of unresectable hepatocellular carcinoma by the U.S.’s FDA and are approved for the treatment of hepatic neoplasia in the EU and Canada [30].

Generally, patients with HCC that are considered for SIRT have unresectable conditions, a life expectancy of at least 3 months, and are ambulatory/capable of self-care (a score less than or equal to 2 according to the Eastern Cooperative Oncology Group, ECOG [27]. On the other hand, contraindications for SIRT are determined by the potentially irreversible radiationinduced organ damage. For instance, significant and uncorrectable blood flow (either by angiogram or pretreatment Tc-99m MAA scan) to the gastrointestinal tract may cause severe gastric or intestinal ulcer and even hollow-organ perforation. Excessive shunting to lungs that can potentially exceed the 30-Gy lung dose limit noted by Tc-99m MAA scan may induce radiation pneumonitis. A large tumor burden with limited hepatic reserve or biochemical evidence of reduced liver function may elicit the post-therapeutic liver failure. Prior external beam radiotherapy involving the liver may increase the possibility of radiationinduced liver disease due to accumulated radiation doses [13].

Although SIRT with ^90^Y-bearing microspheres has an acceptable safety profile, studies comparing ^90^Y-bearing microspheres to other palliative loco-regional therapies and thus consensus about the optimal use of ^90^Y-bearing microspheres are currently lacking [31]. However, SIRT with ^90^Y-bearing microspheres, especially with the glass-based microspheres, because of their low embolic potential, may be preferred to transarterial chemoembolization (TACE) in the presence of portal vein occlusion [32-34]. SIRT is also not currently listed among possible treatment options for HCC in some guidelines [35]. However, there have been suggestions to use SIRT as a bridging treatment or as a main therapy for patients with diffuse intrahepatic tumor involvement [36]. Nevertheless, the therapy may be considered for patients with an insufficient hepatic reserve, poor performance status, and the comorbidities of a resectable disease [37]. Potential indications for specific clinical conditions are listed in the following paragraphs.

### 4.1. Bridging therapy for subsequent liver transplantation

Patients with early-stage hepatocellular carcinoma can be candidates for liver transplantation as a curative treatment. However, organ donors, compared to those in need of organs, are few and far between, causing long waiting list for transplantation. Hence, patients in need of a liver transplant may be at a high risk for disease progression, which would subsequently render them ineligible for transplantation surgery. To decrease the probability of disease progression while waiting, these patients are usually treated with various kinds of locoregional therapies, such as TACE and percutaneous ablation, with the aim of temporarily restricting the tumor growth. It is in this context that SIRT has also been proposed for theoretically being better able to control tumor growth and disease progression as compared to the aforementioned traditional approach. However, SIRT has not been widely applied and validated due to its relatively high cost [38].

### 4.2. Downsizing/downstaging therapy for subsequent curative surgery

For patients with intermediate-stage hepatocellular carcinoma, shrinkage of the tumor may convert the initially unresectable tumor to a resectable tumor, or perhaps put the patient in an eligible condition for liver transplantation [39]. The SIRT method has been compared to the traditional TACE method regarding its effect on downsizing/downstaging tumors to allow for liver transplantation, and the data have shown a higher percentage of SIRT patients with tumor downstaging (58%, compared to 31% of TACE) [40].

### 4.3. “Radiation lobectomy” to induce hypertrophy of liver remnant for subsequent curative surgery

Portal vein embolization is a standard technique for patients with liver malignancies that are not amenable to surgical resection owing to the small liver remnant that would remain after surgery. Portal vein embolization induces contralateral hypertrophy by redirecting the portal blood flow, which would increase the volume of the post-surgery liver remnant [41, 42]. However, there has been concern raised about the progression of diseases left untreated while waiting for the hypertrophy process to have its effect [43]. Recently “radiation lobectomy”, defined as the transarterial lobar infusion of ^90^Y-bearing microspheres, has been found to induce similar or superior volumetric changes as portal vein embolization in hepatic lobes. It induces marked hypotrophy of the treated hepatic lobe and evident hypertrophy of the contralateral lobe. By limiting the rate of portal blood flow diversion, this therapy also effectively treats the cancer itself and reduces the risk of preinterventional tumor progression [42, 44].

### 4.4. Local control and palliative therapy for inoperable disease

In a retrospective analysis consisting of 122 HCC-affected patients treated with TACE and 123 HCC-affected patients treated with glass-based SIRT, the overall survival of patients in all-stages (median survival of TACE and SIRT are 17.4 and 20.5 months, respectively, *P* = 0.232) or in the intermediate-stage (17.2 and 17.5 months, respectively, *P* = 0.42) were found to be similar [45]. However, significantly more frequent fatigue, nausea, anorexia, and abdominal pain were observed in patients treated with TACE, suggesting a more favorable safety profile and a potentially reduced need for hospitalization to treat adverse effects with SIRT treatment [46-49].

## 5. Safety of repeated selective internal radiation therapies

Repeated selective internal radiation therapies of liver tumors theoretically create a higher risk of developing radiation-induced liver disease because of the accumulated unrecoverable injuries done to the liver. Lam *et al*. retrospectively analyzed 8 patient treated by repeated selective internal radiation therapies with resin-based microspheres and the BSA method [50]. Two of them died shortly after the second treatment (at 84 and 107 days), and their deaths were attributed to radiation-induced liver disease. Both patients underwent whole liver treatment twice (cumulative activities of 3.08 and 2.66 GBq), and were heavily treated with multiple systemic chemotherapy and tumor resections before the 1^st^ SIRT and between two selective internal radiation therapies. The authors concluded that repeated SIRT of the same targeted liver volume, especially in whole liver treatments and high cumulative activities, might increase the risk for development of radiation-induced liver disease.

On the other hand, Zarva *et al*. performed repeated SIRTs to 21 patients with advanced liver tumors [51]. The authors also used resin-based microspheres and the BSA method for treatments. However, they treated patients only 1 lobe at a time, and there was an interval between selective internal radiation therapies of both lobes of 4 to 6 weeks if both liver lobes needed to be treated in a single SIRT cycle. An average of 1.6 whole-liver treatments in 3.0 unilobar selective internal radiation therapies (liver lobes sequentially) were given to patients with a mean total activity of 2.57 GBq. No radiation-induced liver disease was observed in any of their patients. They concluded that there was a significantly better tolerance to SIRT in patients with sequential lobar treatments than in patients receiving single-session wholeliver treatments.

## 6. Safety of sirt after ebrt

There is limited literature discussing the safety of performing SIRT after EBRT. In one retrospective study consisting of 31 patients, dose-volume analysis of the liver showed that the fraction of liver exposed to ≥ 30 Gy (V30) was the strongest predictor of hepatotoxicity [52]. Patients with V30 > 13% had hepatotoxicity, and fatal radiation-induced liver disease occurred in two patients with the highest mean liver doses of EBRT (20.9 Gy and 23.1 Gy) and also at the highest cumulative liver doses (91.8 Gy and 149 Gy). Hence, SIRT may be safe only for patients with limited hepatic exposure to previous EBRT, and should be used cautiously if the prior V30 of the liver exceeded about 10%.

## 7. Conclusions

SIRT with ^90^Y-bearing microspheres offers an opportunity for effective local tumor control and even tumor regression in patients with unresectable hepatocellular carcinomas. Moreover, it causes less adverse effects as compared to TACE, as well as decreasing the need for prolonged hospitalization. Radiation lobectomy seems promising as an alternative technique to portal vein embolization for patients with liver malignancies not amenable to surgical resection owing to small liver remnant after surgery. However, more evidence from prospective studies among patients with HCC at various stages are necessary to confirm the usefulness of SIRT.
